# Blood biomarkers in male and female participants after an Ironman-distance triathlon

**DOI:** 10.1371/journal.pone.0179324

**Published:** 2017-06-13

**Authors:** Tom Danielsson, Jörg Carlsson, Hendrik Schreyer, Jonas Ahnesjö, Lasse Ten Siethoff, Thony Ragnarsson, Åsa Tugetam, Patrick Bergman

**Affiliations:** 1 Department of Sport Sciences, Linneaus University, Kalmar, Sweden; 2 Department of Biosciences, Linneaus University, Kalmar, Sweden; 3 Department of Cardiology, Kalmar Hospital, Kalmar, Sweden; 4 Department of Pedagogics, Linneaus University, Kalmar, Sweden; Texas A&M University, UNITED STATES

## Abstract

**Background:**

While overall physical activity is clearly associated with a better short-term and long-term health, prolonged strenuous physical activity may result in a rise in acute levels of blood-biomarkers used in clinical practice for diagnosis of various conditions or diseases. In this study, we explored the acute effects of a full Ironman-distance triathlon on biomarkers related to heart-, liver-, kidney- and skeletal muscle damage immediately post-race and after one week’s rest. We also examined if sex, age, finishing time and body composition influenced the post-race values of the biomarkers.

**Methods:**

A sample of 30 subjects was recruited (50% women) to the study. The subjects were evaluated for body composition and blood samples were taken at three occasions, before the race (T1), immediately after (T2) and one week after the race (T3). Linear regression models were fitted to analyse the independent contribution of sex and finishing time controlled for weight, body fat percentage and age, on the biomarkers at the termination of the race (T2). Linear mixed models were fitted to examine if the biomarkers differed between the sexes over time (T1-T3).

**Results:**

Being male was a significant predictor of higher post-race (T2) levels of myoglobin, CK, and creatinine levels and body weight was negatively associated with myoglobin. In general, the models were unable to explain the variation of the dependent variables. In the linear mixed models, an interaction between time (T1-T3) and sex was seen for myoglobin and creatinine, in which women had a less pronounced response to the race.

**Conclusion:**

Overall women appear to tolerate the effects of prolonged strenuous physical activity better than men as illustrated by their lower values of the biomarkers both post-race as well as during recovery.

## Introduction

While overall physical activity is clearly associated with better short-term and long-term health [1], prolonged strenuous physical activity may result in a rise in various biomarkers for example; cardiac biomarkers typically seen with myocardial infarction, cardiac troponin t (cTnT) and N-terminal of the prohormone brain natriuretic peptide (NT-proBNP) [2–4], myoglobin which indicates breakdown of skeletal muscle [5–7], creatinine signalling kidney damage [7–10], and alanine aminotransferase (ALT) and aspartate aminotransferase (AST) which in a clinical setting would be indicative of liver overload [11, 12]. Inflammation, oxidative stress and DNA damage [7, 13–17] have been seen following prolonged strenuous physical work. Typically in clinical practice, the higher the value of a biomarker, the greater the severity of a given condition or a disease, and it is plausible that higher post-race values are associated with a risk of negative health consequences later in life. However, the importance of the acute response to sporting events involving prolonged strenuous physical activity is debated.

Two competing hypotheses suggest that either the response is a transient harmless normal response to the activity [18], or that repeated ultra-endurance races may in the long term cause negative health consequences, such as atrial fibrillation [19]. There is some evidence supporting the latter, as a J- or U-shaped dose-response curve has been seen between habitual physical activity and morbidity [20]. Knowing what underlying factors are correlated to the magnitude of change in the biomarkers would enable better understanding of the physiological response of a prolonged strenuous physical activity event for amateur participants. This would benefit both health professionals as well as the athletes themselves.

Over the past four decades, participation in endurance events, like triathlon, has grown dramatically, particularly among women [21]. However, little is known regarding the impact of these events on the health of the participating women. In particular there is a death of information regarding how these events effect the aforementioned biomarkers. Therefore, the purpose of this study were to examine the acute effects of a full Ironman-distance triathlon on biomarkers related to heart-, liver-, kidney-, and skeletal muscle damage immediately post-race and one week post-race; the Ironman-distance triathlon is classed as an ultra-endurance event and encompasses a 3.8 km swim, 180 km cycle followed by a 42 km run. We also examined if sex, age, finishing time and body composition influenced the post-race values of the biomarkers.

## Methods

### Ethical considerations

This pilot study received ethical approval from the South-east ethical committee, and ethical approval was also obtained from the regional ethical committee in Linköping (Dnr 2016/86-31). A signed informed consent was obtained from all participants.

### Study sample

Thirty subjects, 15 males and 15 females, who had signed up to participate in an Ironman triathlon in a town in Sweden, were recruited from local sport clubs associated with the Swedish Triathlon Federation. All but one participant successfully completed the full triathlon. Since our sample was not selected at random there is a risk of selection bias. Therefore we tested, using a one sample t-test, if the sample differed with respect to age and finishing time from the overall population of Ironman participants (i.e. average obtained from 41,000 finishers [22]). There were no significant differences in either age (p = 0.590) or finishing time (p = 0.780).

### Procedure

Prior to the race the subjects were measured for weight and body composition using bioelectrical impedance analysis (BIA) on a Tanita BC-545 Body Composition Analyzer (Tanita, Inc., Tokyo, Japan). Height was measured on a SECA 217 stadiometer (SECA medical measurements and scales, Hamburg, Germany). All participants also filled out a questionnaire containing questions regarding gender, year of birth, number of completed Ironman competitions, year of first race, medications before and during the race, and number of training hours per week during the last year.

Blood samples were drawn from an antecubital vein, approximately two weeks before the event (T1), within one hour of finishing (T2) and approximately one week after the race finished the participants returned to the local hospital for the final blood sample (T3). The samples were transferred to the local hospital for analysis of blood biomarkers within three hours after they were drawn.

### Biomarker analysis

Before transfer to the laboratory, a droplet of blood was taken from the blood sample tubes for on-site analyses of number of white blood cells (WBC) using the WBC diff system (Hemocue AB, Sweden).

Blood samples were analysed for the following; cTnT, NT-proBNP, ALT, AST, creatinine, myoglobin and creatin kinase (CK). cTnT, NT-proBNP and myoglobin were analysed using a electrochemiluminiscence immunoassay on automated analysers (Cobas e411, Roche Diagnostics GmbH, Mannheim, Germany) in the laboratory of Kalmar Hospital. ALT, AST and CK were analysed using the multiple-point (and creatinine the two-point) dry chemistry method (Vitros 5.1 FS Ortho Clinical Diagnostics, Ortho-Clinical Diagnostics, Johnson-Johnson Company, Rochester, USA). The laboratory methods used are standard methods used by the Kalmar Hospital and are validated according to Sweden's national accreditation body, Swedac (www.swedac.se).

### Statistics

All analyses were performed using IBM SPSS version 23. Descriptive data are presented as mean ± standard deviation (SD). Sex differences of the baseline values were analysed using an independent samples t-test.

To investigate the influence of age, sex, finishing time, body fat percentage or body weight on the values at T2, we fitted a series of linear regression models on the biomarkers at completion of the race. To satisfy the assumption of normally distributed residuals and/or homoscedasticity of the residuals, cTnT, myoglobin, CK, AST and creatinine were log-transformed. The models showed no signs of multicollinearity.

To examine if the sexes differed in their responses at T2 and T3, a series of linear mixed models were fitted. We entered the person as subject variable and as repeated measure the time of measurement. Sex and time of measurement (T1-T3) and their interaction term (sex*time) were the fixed factors and the biomarkers were entered as dependent variables. In the models we assumed a diagonal covariance matrix.

## Results

Descriptive data of the studied sample is shown in Table 1. One woman did not complete the race and was excluded from all analyses.

**Table 1:** Descriptive data for the study sample and the biomarkers at baseline (T1). P-values are from independent samples t-test. Clinical reference (Ref) values are provided.

	Total (n = 29)	Men (n = 15)	Women (n = 14)	
	Mean ± SD	Mean ± SD	Mean ± SD	p
Height (cm)	174.8±9.5	181.7 ± 7.2	167.9 ± 5.7	<0.001
Weight (kg)	70.7±12.8	78.4±12.6	62.9±7.2	<0.001
Body fat (%)	15.5±6.1	11.1±4.1	19.9±4.5	<0.001
Age (y)	42.5±6.5	45.1±3.6	40.1±7.6	0.036
Body Mass Index (kg*m^-2^)	23.0±2.5	23.7±2.7	22.3±2.2	0.143
Finishing time (min)	750±92	712±85	791±83	0.017
NT-proBNP (Ref <300 ng/L)	77.8±54.4	60.1±25.2	95.5±69.5	0.034
cTnT (Ref <15 ng/L)*	7.8±4.6	8.5±4.3	7.1±4.9	0.208
Myoglobin (Ref <58 ug/L)*	44.4±29.7	58.3±35.9	30.6±11.0	0.008
CK (Ref <3.6 ukat/L)*	2.6±1.9	3.7±2.1	1.5±0.7	<0.001
AST (Ref <0.61 ukat/L)*	0.6±0.1	0.6±0.1	0.5±0.1	0.018
ALT (Ref <0.76 ukat/L)	0.6±0.2	0.6±0.2	0.5±0.1	0.260
WBC (Ref 3,5–8,8 *10^9^/L)	7.0±2.7	6.5±2.1	7.5±3.1	0.276
Creatinine (Ref 50–90 umol/L)	80.2±14.2	87.4±11.4	73.0±13.4	0.004

*Indicates that the analyses were performed on log-transformed data.

The association between the values of the biomarkers post-race (T2) and sex, weight, %BF, age, and finishing time is summarized in Table 2. Being male was a significant predictor of higher myoglobin (p = 0.005), CK (p = 0.007) and creatinine (p<0.001) levels. Weight was negatively associated with myoglobin (p = 0.033). In general the models were unable to explain the variation of the dependent variables as indicated by the low R^2^-values, with the exception of creatinine, which could be explained to a large extent by the model (R^2^ = 52.8%). For ALT, the coefficient of determination was negative.

**Table 2:** The outcome of the linear regression models.

	WBC R^2^ = 5.2%		NT-proBNP R^2^ = 2.1%		Troponin T* R^2^ = 16.5%		Myoglobin* R^2^ = 21.9%		CK* R^2^ = 22.1%		AST* R^2^ = 13.0%		ALT R^2^ = -3.2%		Creatinine* R^2^ = 52.8%	
	b	p	b	p	b	p	b	p	b	p	b	p	b	p	b	p
Sex (0 = female, 1 = male)	2.80	0.285	188.18	0.562	0.68	0.251	1.82	0.005	1.82	0.007	0.84	0.014	0.176	0.280	0.55	<0.001
Weight (kg)	-0.07	0.376	-12.48	0.177	-0.03	0.137	-0.04	0.033	-0.03	0.112	-0.02	0.096	-0.002	0.659	-0.01	0.173
Total BF (%)	0.21	0.294	22.79	0.355	-0.02	0.635	0.06	0.168	0.06	0.224	0.03	0.222	-0.001	0.965	0.02	0.122
Age (year)	-0.11	0.332	-7.19	0.621	-0.02	0.448	0.01	0.841	-0.02	0.430	0.00	0.785	0.004	0.622	0.00	0.758
Finishing time (h)	-1.12	0.057	7.42	0.916	-0.06	0.658	0.01	0.934	0.20	0.145	0.08	0.245	0.036	0.304	-0.03	0.341

*Indicates that the analyses were performed on log-transformed data.

**Fig 1:**
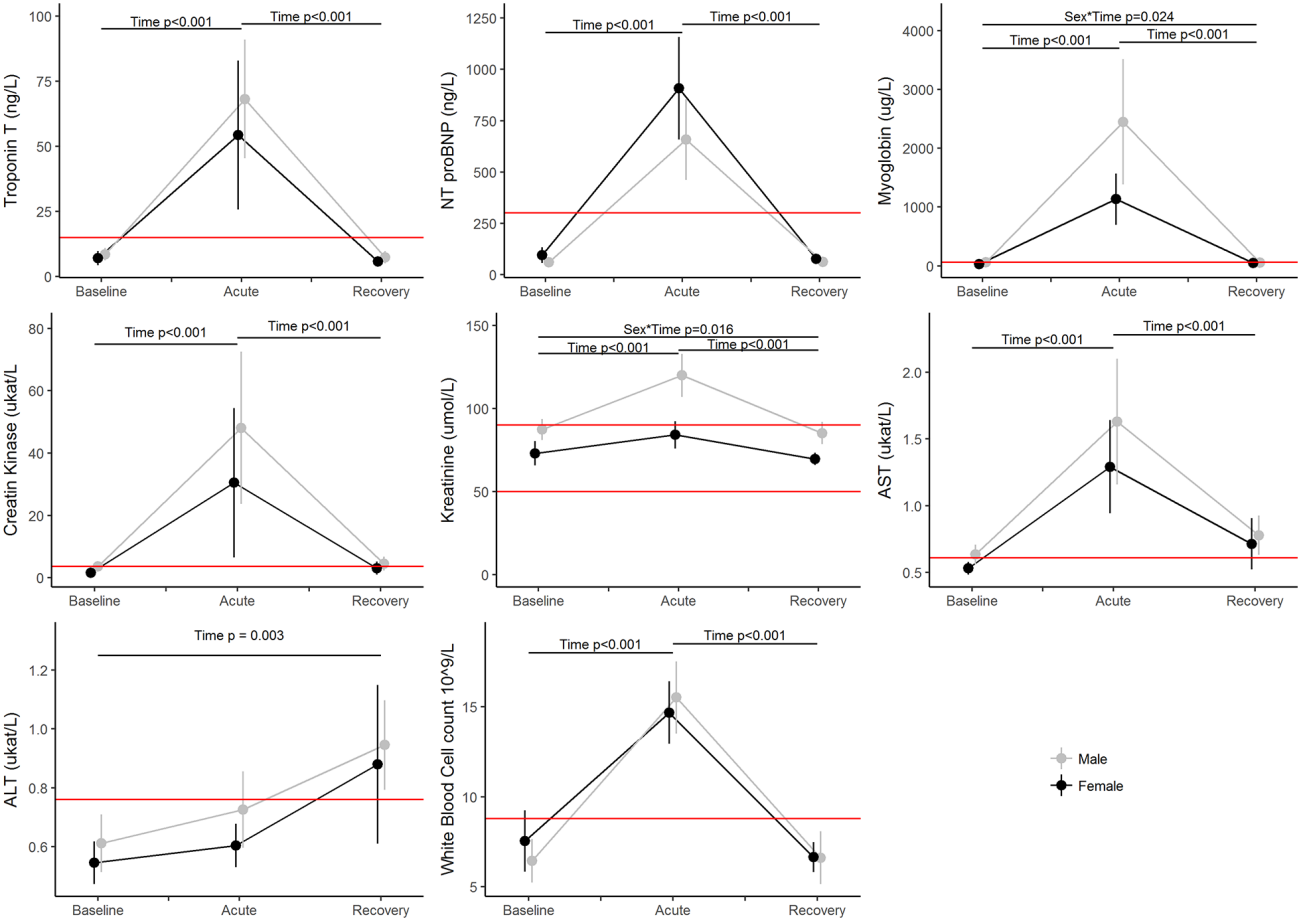
Mean and 95% confidence intervals of the biomarkers by sex from baseline (T1) to recovery (T3). The horizontal lines indicate clinical reference values. The p-values are from the linear mixed models.

As seen in Fig 1, all values at T2, with the exception of ALT, increased significantly (p<0.001) compared to T1. Furthermore, all of the values were well above the clinical reference values. Myoglobin represents the most extreme value in this regard, with the average value rising to 31 and 17 times the clinical reference point for males and females respectively. The level of ALT were highest at T3 and differs significantly from the baseline value (p = 0.003).

An interaction between sex and time of measurement was seen for myoglobin (p = 0.024) and creatinine (p = 0.016) in which the males displayed a higher increase for the post-race levels (T2) compared to the females.

## Discussion

In this study, we have explored the acute effects on biomarkers related to heart-, liver-, kidney- and skeletal muscle damage in a sample of amateur athletes after a full Ironman-distance triathlon. Specifically, women appeared to tolerate the adverse effects of prolonged strenuous physical activity better than men, as illustrated by their lower values of these biomarkers both immediately post-race as well as one week post-race.

The magnitude of the changes from baseline to the post-race measurement was higher in our study compared to most other comparable studies. For example, we have previously reported that in all but one subject, cTnT reached levels in the range of that indicative of myocardial infarctions [23]. This is far higher than reported in other studies [24]. However, the underlying causes to the variation in biomarker response are to a large extent unknown. In this study we examined if sex, age, body weight, body fat percentage or finishing time influenced on the changes. We show that sex is a significant predictor, on both the post-race values of Myoglobin, CK, AST and Creatinine and during the recovery (T1-T3) for Myoglobin and Creatinine, where the women displayed lower values. This finding has not, to our knowledge, previously been reported. In fact, evidence from other ultra-endurance races as well as experimental evidence suggests no sex difference in skeletal muscle damage [25, 26]. It could be hypothesised that the body composition among women with relatively lower muscle mass compared to men will cause the women to suffer less from muscle breakdown. However, we adjusted for both body weight and body fat percentage in the models which suggest that differences in body composition could not explain the difference. Estrogen protects against skeletal muscle breakdown [27] and could be one potential explanation to why women show less skeletal muscle breakdown. But If oestrogen would play a pivotal role in the protection against skeletal muscle damage after ultra-endurance races our observation would be more similar to those previously reported [25, 26].

Overall, with the exception of creatinine, our models explained very little variance, ranging from a few percent up to 20%, indicating that factors other than those included in this study may contribute to the biomarker response following an Ironman triathlon. There are some signs that the intensity of the effort and mechanical stress are two such unmeasured factors. The relative intensity is correlated with cTnT release such that higher intensity but shorter duration leads to a greater release [28, 29]. In this study we used finishing time as a measure of the performance of the athletes but it may be a too crude a measure of the intensity. Two subjects can have the same finishing time but may have used different pacing strategies and thus been working on very different intensities during the race.

To examine the importance of relative intensity we should have fitted the athletes with heart rate monitors. This factor should be considered in future research. There is considerable mechanical stress associated with running (one of the components of the triathlon), where each step sends a shock wave (i.e. ground reaction force) of 2–3 times the body weight through the body [30]. These forces have been linked to skeletal muscle injury and may also be a contributing factor to several of the other biomarkers as well. For example, we saw high values of myoglobin, CK and AST. This “cluster” is related to the breakdown of skeletal muscle and is a sign of exertional rhabdomyolysis [31]. Furthermore, this could be one of the reasons why completing a full marathon (42 km) leads to higher cTnT values compared to long cycle races [24]. Measuring the ground reaction force during the marathon part would enable us to investigate the mechanical stress as a predictor of the acute levels of blood biomarkers. This could be done by equipping the athletes with accelerometers which have been shown to be able to measure the ground reaction force [32].

The clinical importance of the findings in this study and those found in many other studies has been debated. The debate centres on if the elevated biomarkers seen after ultra-endurance races reflect true pathological changes or if they are a part of normal physiological processes that occurs due to adaptation [18, 19]. Most of the debate concerns the release of cTnT into the blood after strenuous prolonged physical activities. Given that cTnT is a relatively small molecule (< 40 kDa) that leaks into the blood stream even after rather modest efforts such as after one hour of spinning [33] or after two standard floor ball games [34] it may not be the best biomarker to study in this context. Therefore, there is a need to identify other biomarkers that may increase the specificity between true pathology and adaptation. In both men and women we saw high post-race values but could not find any significant difference in their response of cTnT or NT-proBNP. However, the women showed non-significant lower values of cTnT and in the long run even such a modest difference could be of clinical importance. It appears as women who are high intensity exercisers have a reduced risk of developing atrial fibrillation later in life while high intensity exercising men have an increased risk [35].

## Conclusion

In this study we have explored the acute effects of a full Ironman-distance triathlon on biomarkers related to heart-, liver-, kidney- and skeletal muscle damage in a sample of amateur athletes. Large acute elevations of the studied biomarkers were seen post-race. These changes were more pronounced among the males indicating that the women tolerate prolonged strenuous physical activity better compared to men. Our models were not able to explain the variation of the studied biomarkers which means that a lot of unmeasured confounding, by as yet-unidentified predictors, is present. Moreover, it is likely, that our sample size was simply too small to yield enough statistical power to adequately model the data. Accepting this, future studies are needed that identify other markers that may increase the specificity between true pathology and physiological adaptation. In the absence of this knowledge and epidemiological data on this population, the clinical relevance of these acute changes after an Ironman race remain unclear.
